# Overlapping Toxicities of Pembrolizumab and Lenvatinib: A Case of Capillary Leak Syndrome with Severe Erythroblastosis

**DOI:** 10.3390/curroncol33050268

**Published:** 2026-05-06

**Authors:** Aikaterini Gkoufa, Iraklis Patsialos, Christos Stafylidis, Amalia Anastasopoulou, Dimitra Adamou, Helen Gogas, Panagiotis T. Diamantopoulos

**Affiliations:** 1 First Department of Internal Medicine, Laiko General Hospital, National & Kapodistrian University of Athens, 11527 Athens, Greece; amanast@med.uoa.gr (A.A.); hgogas@med.uoa.gr (H.G.); 2 Hematology Unit, First Department of Internal Medicine, Laiko General Hospital, National & Kapodistrian University of Athens, 11527 Athens, Greece; irpatsialos@med.uoa.gr (I.P.); christosstaf@med.uoa.gr (C.S.); diamp@med.uoa.gr (P.T.D.); 3 Hematology Laboratory, Laiko General Hospital, 11527 Athens, Greece; dadamou@gmail.com

**Keywords:** capillary leak syndrome, lenvatinib toxicity, hematologic toxicity, melanoma, immune-checkpoint inhibitors

## Abstract

Combined immune checkpoint inhibition (ICI) and multitargeted tyrosine kinase inhibition (TKI) improve outcomes in advanced melanoma, especially in heavily pretreated patients, but introduce complex, overlapping toxicities. Although immune-related adverse events and TKI-specific toxicities are well characterized individually, their concurrent presentation is uncommon and can obscure diagnosis. Capillary leak syndrome (CLS), a rare but potentially life-threatening complication of ICIs, further complicates recognition due to nonspecific features and variable onset. A 43-year-old woman with metastatic BRAF wild-type melanoma, in complete metabolic response on lenvatinib and pembrolizumab, presented with generalized edema, hypotension, thrombocytopenia, and marked erythroblastosis. Extensive evaluation, including bone marrow analysis, excluded malignancy, infection, and autoimmune disease, but revealed multilineage dysplasia with pronounced erythroid stress. Imaging confirmed sustained remission with pleural effusions and ascites. Dual toxicity was suspected: lenvatinib-related hematologic toxicity and pembrolizumab-associated CLS. Both agents were discontinued, and corticosteroids followed by IVIG for steroid-refractory edema led to gradual recovery. This case underscores the diagnostic challenge of overlapping ICI–TKI toxicities. Mechanistically, VEGF pathway inhibition may disrupt marrow endothelial integrity and hematopoietic homeostasis, promoting cytopenias and erythroid precursor release, while immune activation drives cytokine-mediated endothelial dysfunction and vascular hyperpermeability. Early recognition and prompt immunomodulatory management are critical to improving outcomes.

## Simple Summary

Combined immune checkpoint inhibitors (ICI) and tyrosine kinase inhibitors (TKI) improve outcomes in advanced melanoma but can cause overlapping toxicities that complicate management. We report a 43-year-old woman with metastatic BRAF wild-type melanoma in complete metabolic response on lenvatinib and pembrolizumab who developed generalized edema, hypotension, thrombocytopenia, and marked erythroblastosis. Extensive investigations, including bone marrow analysis, excluded malignancy, infection, and autoimmune causes, but showed multilineage dysplasia with erythroid stress. Imaging confirmed disease remission but pleural effusions and ascites. A dual toxicity was suspected: lenvatinib-induced hematologic toxicity and pembrolizumab-associated capillary leak syndrome (CLS). Treatment discontinuation, corticosteroids, and intravenous immunoglobulin (IVIG) for steroid-refractory edema led to gradual recovery. This case highlights the diagnostic complexity of combined ICI–TKI toxicities. Mechanistically, vascular endothelial growth factor (VEGF) inhibition may impair marrow endothelial integrity and hematopoiesis, while immune activation drives cytokine-mediated endothelial dysfunction and vascular hyperpermeability, emphasizing the importance of early recognition and timely immunomodulatory management.

## 1. Introduction

Immune checkpoint inhibitors (ICIs) and multitargeted tyrosine kinase inhibitors (TKIs) have transformed the therapeutic landscape of metastatic melanoma. Their combination has so far demonstrated encouraging activity, particularly in patients who have previously received multiple lines of therapy and has demonstrated encouraging efficacy across multiple tumor types [1,2]. However, their use is frequently complicated by immune-related adverse events (irAEs) and drug-specific toxicities and, although ICIs and TKIs are each associated with distinct toxicity profiles, the simultaneous occurrence of clinically relevant adverse events attributable to both treatments in the same patient remains uncommon.

Lenvatinib, a multitargeted receptor TKI that inhibits VEGFR1-3, FGFR1-4, PDGFRA, c-KIT, and RET, is increasingly employed in advanced melanoma resistant to initial ICI therapy, and particularly in heavily pretreated patients. ICIs are well known to induce a wide spectrum of irAEs, ranging from mild to severe, and potentially affecting any organ system [3,4,5]. In this manuscript, we describe a case complicated by a constellation of adverse events related to lenvatinib and pembrolizumab, a phenomenon that remains infrequently reported, underscoring the clinical importance of distinguishing overlapping and agent-specific toxicity mechanisms during combination treatment.

## 2. Case Presentation

A 43-year-old woman with metastatic melanoma (stage IV, BRAF wild-type) presented to the emergency department of Laikon General, a tertiary care center in Athens, Greece, due to severe headache, tinnitus, progressive thrombocytopenia, and generalized edema. She had no other notable comorbidities and was not on chronic medication, apart from occasional use of oral iron supplementation. The primary melanoma lesion had been diagnosed thirteen years earlier and was managed with surgical excision, followed by routine imaging surveillance. Four years before the current presentation, disease progression was documented and the patient received first-line treatment with the combination of nivolumab and ipilimumab and, after achieving stable disease, she was transitioned to maintenance therapy with nivolumab, which was continued for seventeen months until radiological evidence of progression. The treatment was complicated by overt eosinophilia, which, after thorough investigation, was attributed to nivolumab. At progression, combination therapy with lenvatinib at 10 mg once daily and pembrolizumab at 200 mg every 21 days was initiated as a palliative treatment option, resulting in a remarkable complete and durable metabolic response (CMR) one year after treatment initiation. Treatment was discontinued one month before admission due to grade 3 eosinophilia, attributed to melanoma therapy and treated with methylprednisolone at 0.75 mg/kg/day, followed by a rapid taper, along with bilastine 75 mg twice daily. Upon admission, the physical examination revealed orthostatic hypotension, pitting soft tissue edema involving the face, arms, forearms, lumbar region, and bilateral ankles, without inflammation, and diminished breath sounds in the lower lobes of both lungs. Initial laboratory investigations revealed grade 1 normocytic anemia, grade 4 thrombocytopenia, grade 2 hypoalbuminemia, and low immunoglobulins (Table 1).

**Figure 1:**
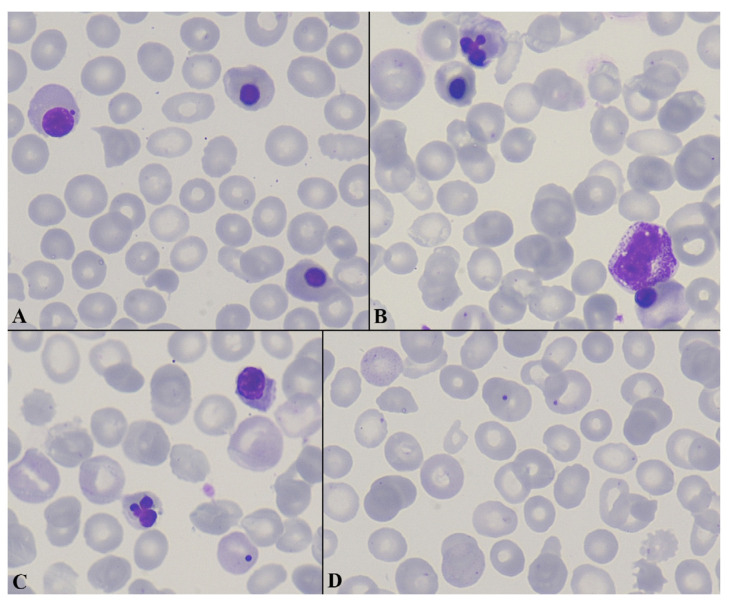
Peripheral blood smear findings (**A**–**D**). Peripheral blood smear (May–Grünwald–Giemsa stain, ×1000) demonstrating marked anisopoikilocytosis with irregularly shaped red blood cells (RBCs), basophilic stippling, polychromasia, and Howell–Jolly bodies. Numerous circulating nucleated red blood cells (NRBCs) are present, representing different stages of erythroid maturation, consistent with erythroblastemia, along with prominent dyserythropoiesis. Occasional immature leukocytes and marked thrombocytopenia are also present.

Peripheral blood smear examination showed a remarkable erythroblastosis with 55 nucleated red blood cells (NRBCs) per 100 white blood cells (WBCs), multiple RBC morphological abnormalities (anisocytosis, poikilocytosis, polychromasia, spherocytosis, schistocytosis, basophilic stippling, Howell–Jolly bodies, and rare tear-drop cells), and severe thrombocytopenia (Figure 1A–D).

A chest X-ray and an abdominal ultrasound demonstrated bilateral pleural effusions with associated lower lobe atelectasis and moderate ascites, borderline splenomegaly with mild parenchymal heterogeneity and preserved vascular flow. An 18-FDG PET/CT scan revealed diffuse, mildly increased uptake throughout the bone marrow, and complete remission of the underlying melanoma. A brain MRI revealed a pansinusitis that was empirically treated with moxifloxacin, though without improvement of the headache.

**Figure 2:**
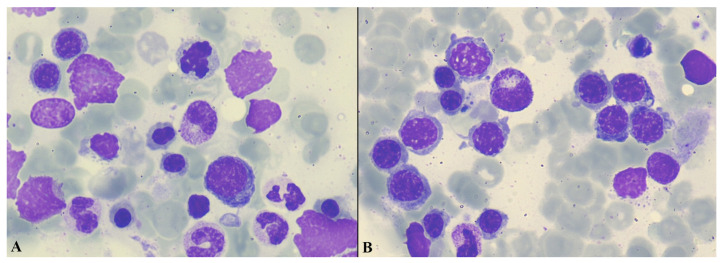
Bone marrow aspirate smear (**A**,**B**). Bone marrow aspirate (May–Grünwald–Giemsa stain, ×1000) demonstrating marked erythroid hyperplasia with a predominance of erythroid precursors at various stages of maturation. Numerous erythroblasts with prominent dysplastic features are present, including early and late forms, some arranged in clusters, along with frequent naked erythroid nuclei. Background myeloid elements are relatively decreased.

An extensive laboratory evaluation for infectious and autoimmune etiologies yielded negative results (Table 1). During hospitalization, there was a marked increase in the number of peripheral blood NRBCs (288 per 100 WBC), along with persistent severe thrombocytopenia. A hemoglobin electrophoresis indicated a normal adult hemoglobin pattern. Examination of a bone marrow smear revealed erythroid hyperplasia, striking dyserythropoietic features in over 10% of erythroid precursors, a low blast percentage (<1%), and no abnormal infiltrate (Figure 2A,B).

Immunophenotyping of the marrow cells confirmed the low blast percentage, and bone marrow cytogenetic analysis revealed a normal female karyotype (46, XX). Periodic acid–Schiff (PAS) staining revealed diffuse but heterogeneous positivity of erythroblasts, with some showing fine granular positivity superimposed on the diffuse pattern. Importantly, no large “lake-like” PAS-positive accumulations typical of MDS and M6 acute myeloid leukemia were observed. Prussian blue staining of the bone marrow showed reduced marrow iron stores, significantly decreased intracytoplasmic iron, and no ring sideroblasts. A Technetium-99m (99mTc) heat-damaged red blood cell (RBC) scintigraphy showed homogeneous radiotracer uptake at the anatomical location of the spleen, with no pathological uptake elsewhere. Splenic sequestration of RBCs was observed, consistent with splenomegaly, but without indications of extramedullary hematopoiesis at ectopic sites.

A 24 h urinary protein quantification excluded albuminuria. The hepatic and cardiac function were comprehensively evaluated using laboratory testing and imaging studies; no abnormalities were identified (Table 1). Diagnostic paracentesis of both pleural and peritoneal effusions revealed no cytologic or immunophenotypic evidence of malignancy. Pleural fluid analysis was consistent with a lymphocyte-predominant exudate according to Light’s criteria [6]. Ascitic fluid analysis showed a low serum–ascites albumin gradient (SAAG < 1.1), supporting a non-portal hypertensive etiology. There was no evidence of protein-losing enteropathy, and thyroid function tests were within normal limits. A skin biopsy demonstrated dermal edema with a mild lymphocytic infiltrate and rare eosinophils, while direct immunofluorescence was non-specific. Plasma C1q levels were also found to be normal. Following exclusion of common causes of generalized edema, and in the presence of concomitant hypotension and headaches, treatment-related CLS was considered the leading diagnosis.

Lenvatinib and pembrolizumab were permanently discontinued due to potentially lenvatinib-induced erythroblastosis and thrombocytopenia, as well as pembrolizumab-associated CLS. The methylprednisolone dose, initially administered for eosinophilia, was escalated to 1 mg/kg/day (prednisolone equivalent), followed by a gradual tapering regimen with dose reductions of 4 mg per week. Furosemide was introduced for symptomatic management of the soft-tissue edema, but due to limited clinical response, IVIG was initiated at a dose of 400 mg/kg (total dose 20 g), resulting in the gradual resolution of the edema. Both the platelet count and hemoglobin levels returned to normal two weeks after high-dose corticosteroids, with no recurrence of thrombocytopenia after lenvatinib discontinuation. Erythroblastosis and dyserythropoietic features also resolved completely two weeks later. Following the observed clinical response, the patient continued treatment with monthly IVIG infusions at the same dose. To date, she has received five treatment cycles, with sustained clinical improvement.

Regarding melanoma management, given the prior achievement of CMR after nearly two years of systemic therapy for the disease, and in light of severe treatment-related toxicities, melanoma-directed pharmacologic therapy was discontinued. The patient remains under close clinical and radiological surveillance, with no clinical or radiological signs of relapse one year after melanoma treatment discontinuation. A detailed chronological timeline of clinical events, treatments, and adverse effects is provided in Table 2.

## 3. Discussion

This case highlights the complex interplay between metastatic melanoma, potential bone marrow infiltration, and TKIs- and ICIs-induced adverse events. Given the constellation of clinical and laboratory findings, including severe thrombocytopenia, multilineage dysplasia, marked erythroblastosis, and evidence of systemic inflammation, a comprehensive diagnostic evaluation was undertaken.

### 3.1. Hematological Toxicity

Following a comprehensive evaluation, as outlined above, all potential causes of the patient’s hematological presentation were systematically excluded, and were presumably attributed to lenvatinib rather than pembrolizumab [7]. VEGF signaling plays a crucial role in maintaining the integrity of the bone marrow vascular niche, which regulates hematopoietic stem cell retention and trafficking. Disruption of this vascular microenvironment may alter the interaction between endothelial cells and hematopoietic progenitors, potentially leading to the mobilization of immature cells into the peripheral circulation. Experimental and translational studies have demonstrated that VEGF signaling contributes to the maintenance of the bone marrow perivascular niche [8,9]. Interference with VEGF-mediated endothelial signaling can disrupt this niche and modify hematopoietic cell trafficking. In addition, VEGF-dependent endothelial permeability is involved in the mobilization of hematopoietic progenitors from the bone marrow into the circulation. Therefore, pharmacologic inhibition of VEGF pathways may theoretically contribute to the release of immature hematopoietic cells, potentially manifesting as erythroblastosis in the peripheral blood. On the other hand, thrombocytopenia is a recognized adverse event of treatment with lenvatinib, and although it is usually mild to moderate, it may be severe. In several studies of lenvatinib in different solid tumors, such as thyroid cancer [10] and hepatocellular carcinoma [11], thrombocytopenia occurred in up to 16% of patients. The mechanism is not completely understood, but it is likely related to its multikinase inhibitory activity. Possible mechanisms include inhibition of the VEGF pathway that disrupts the microenvironment supporting hematopoiesis and megakaryocyte maturation, PDGFR inhibition, which may affect stromal support of megakaryopoiesis, and endothelial injury that may occasionally cause microangiopathic processes contributing to platelet consumption. Overall, the mechanisms underlying lenvatinib-associated hematologic toxicity are not fully elucidated. Nevertheless, several biologically plausible pathways have been proposed. Beyond its role in angiogenesis, VEGF signaling contributes, as already reported, to the regulation of hematopoietic stem cells and megakaryocyte maturation [12]. Pharmacologic inhibition of VEGF receptors by multikinase inhibitors such as lenvatinib may therefore interfere with normal hematopoietic processes, including platelet production [12]. In addition, inhibition of other targets of lenvatinib, such as fibroblast growth factor receptors (FGFR) and c-KIT, which are implicated in progenitor cell proliferation and megakaryocytopoiesis, may further contribute to the development of cytopenias [13]. Despite these considerations, the precise contribution of each pathway remains uncertain, and the mechanisms underlying more complex hematologic findings, including erythroblastosis and dysplastic features, are yet to be clearly defined. The potential mechanisms of erythroblastosis and thrombocytopenia of the present case are depicted in Figure 1. Underlying marrow disease, infection or inflammation, drug-induced marrow suppression, and other causes of severe thrombocytopenia should be evaluated before attributing thrombocytopenia to lenvatinib. On the other hand, pembrolizumab-associated thrombocytopenia is rare, affecting less than 1% of patients, and is usually immune-related [14].

### 3.2. Capillary Leak Syndrome

CLS is characterized by increased capillary endothelial permeability, leading to the extravasation of protein-rich fluid into the interstitial space [15]. Clinically, this process manifests mainly as diffuse edema, exudative serous cavity effusions, and frequently hypotension [16]. CLS may occur as an idiopathic condition or arise secondary to underlying diseases or pharmacologic agents. Despite differences in etiology, the fundamental pathophysiologic mechanism involves disruption of the endothelial barrier integrity, primarily through alterations in intercellular junctions [15]. The major molecule responsible for endothelial stability and regulation of vessel permeability is the vascular endothelial cadherin [17]. This protein ensures its stabilization by interaction with the cytoskeleton, specifically via catenins, vinculin, and other actin-binding proteins, such as epithelial protein lost in neoplasm (EPLIN)-α and -β [18]. In the presence of inflammation and increased cytokines, such as IL-2, IL-6, IL-11, IL-12, and TNF-α, cadherin internalizes into the cells, leading to the disruption of cellular junctions [15]. Moreover, phosphorylation of those proteins, mediated by VEGF, probably further contributes to the increase in vascular permeability [19]. Many factors could potentially lead to endocytosis and degradation of those molecules. Among the pharmacologic regimens implicated in the development of CLS, antineoplastic therapies—and particularly ICIs—have been increasingly recognized in the literature as potential triggers of this condition [20,21]. The cytokine imbalance induced by ICI administration may provide a mechanistic link between these two entities. By releasing inhibitory signals on CD4^+^ and CD8^+^ T cells, ICIs lead to their excessive activation, resulting in increased production of key effector cytokines, mainly IL-2, TNF-α, and IFN-γ. This primary cytokine imbalance subsequently promotes activation of myeloid and endothelial cells, leading to secondary upregulation of pro-inflammatory mediators such as IL-6 and IL-1β [22]. Notably, these cytokines are well-established contributors to endothelial dysfunction and increased vascular permeability, which represent central features in the pathophysiology of CLS. However, this syndrome remains an uncommon clinical entity and its diagnosis can be challenging, since its manifestations are often nonspecific and frequently overlap with those of other conditions characterized by fluid shifts and edema. As a result, timely recognition may be complex and demanding in routine clinical practice. Nevertheless, given the potential for rapid clinical deterioration and life-threatening complications, heightened clinical awareness of the condition remains essential.

Regarding the timing of CLS onset, it is well recognized that ir-toxicities may exhibit delayed onset, emerging many months after immune checkpoint inhibitor administration and, in some cases, even after treatment discontinuation [23,24]. The patient developed hypotension, hypoalbuminemia, generalized edema, and exudative serous cavity effusions, accompanied by symptoms such as headaches and tinnitus, clinical features that have been previously described in association with CLS [15,16,21,25]. Importantly, an extensive diagnostic evaluation excluded alternative etiologies that could plausibly account for the full spectrum of these findings, including cardiac, hepatic, renal, infectious, and autoimmune causes. The presence of inflammatory changes in the paranasal sinuses that were unresponsive to antibiotic therapy raised the suspicion of a potential association with ICI–related inflammation [26]. A potentially misleading finding in the present case was the relatively low hemoglobin levels, given the fact that high hemoglobin levels are considered a typical finding of CLS, due to hemoconcentration. This discrepancy may be explained by the concomitant hematologic toxicity attributed to lenvatinib. Consequently, the patient did not exhibit the complete laboratory pattern commonly described in the literature, highlighting the potential for atypical presentations in the setting of overlapping treatment-related toxicities.

**Figure 3:**
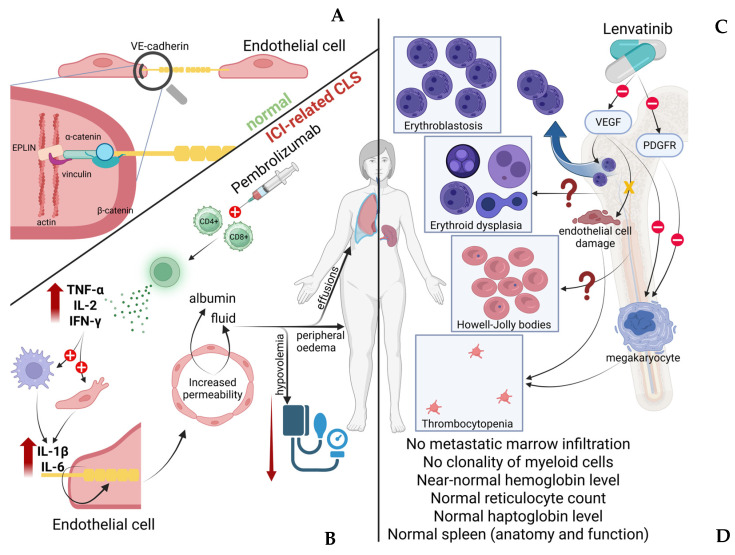
Integrated schematic representation of the proposed mechanisms underlying immune checkpoint inhibitor–related capillary leak syndrome and lenvatinib-associated hematologic abnormalities. **Left panel** (endothelial homeostasis and capillary leak syndrome). (**A**) **Upper-left part.** Normal structure of the endothelial barrier, highlighting the central role of vascular endothelial (VE)-cadherin in maintaining intercellular junction integrity. VE-cadherin interacts with intracellular adaptor proteins, including α- and β-catenin, vinculin, and actin-binding proteins such as EPLIN, thereby stabilizing the endothelial cytoskeleton and preserving vascular integrity. (**B**) Following administration of pembrolizumab, immune checkpoint inhibition leads to activation of CD4^+^ and CD8^+^ T cells, resulting in increased production of pro-inflammatory cytokines, including TNF-α, IL-2, and IFN-γ. Activation of myeloid and endothelial cells induces secondary release of cytokines such as IL-1β and IL-6. The resulting cytokine imbalance causes internalization of cadherin and disruption of endothelial junctions, leading to increased vascular permeability and leakage of plasma components (albumin and fluid) into the interstitial space. Clinically, this manifests as hypovolemia, peripheral edema, and effusions, consistent with immune checkpoint inhibitor (ICI)–related capillary leak syndrome (CLS). **Right panel** (lenvatinib-associated hematologic toxicity). (**C**) The **upper-right side** of the figure summarizes the observed hematologic abnormalities during treatment with lenvatinib. Inhibition of VEGF signaling is hypothesized to disrupt the bone marrow vascular niche, altering interactions between endothelial cells and hematopoietic progenitors. This may lead to the mobilization of immature erythroid precursors into the peripheral circulation, causing erythroblastosis. Additional findings include erythroid dysplasia, the presence of Howell–Jolly bodies, and thrombocytopenia, suggesting impaired erythropoiesis and megakaryopoiesis. Potential contributing mechanisms include endothelial injury, stromal dysfunction, and impaired megakaryocyte maturation. However, the exact pathophysiology remains unclear, as indicated by the question marks in the schematic. (**D**) **Bottom right**. Despite these hematologic abnormalities, key diagnostic features arguing against primary bone marrow and splenic pathology are highlighted. Abbreviations. CLS, capillary leak syndrome; ICI, immune checkpoint inhibitor; VE-cadherin, vascular endothelial cadherin; VEGF, vascular endothelial growth factor; PDGFR, platelet-derived growth factor receptor; EPLIN, epithelial protein lost in neoplasm; TNF-α, tumor necrosis factor alpha; IL-1β/IL-2/IL-6, interleukin-1 beta, interleukin-2, interleukin-6; IFN-γ, interferon gamma; CD4^+^/CD8^+^, cluster of differentiation 4 and 8 positive T lymphocytes.

Furthermore, pembrolizumab was deemed the most probable driver of CLS in this patient for several other reasons. First, an increasing number of published data have documented the occurrence of CLS in association with ICIs, supporting a plausible causal relationship [20,21]. Second, the patient had already been evaluated for eosinophilia before presentation, an ir-AE well described in the context of ICI treatment, suggesting an activated or dysregulated immune milieu [27]. Third, lenvatinib is not typically associated with capillary hyperpermeability; conversely, VEGF pathway inhibition has been explored as a therapeutic strategy for conditions characterized by increased vascular permeability [23,28,29]. Taken together, these observations favored pembrolizumab as the more plausible contributor to CLS in this case. Figure 3 depicts the possible pathophysiological mechanisms underlying ICI–related CLS.

Regarding our management strategy, the initial, critical step involved withdrawal of the suspected offending agents. Importantly, given that these agents are administered as a combined therapeutic regimen for metastatic melanoma, even if toxicity had been attributed primarily to a single drug, continuation of the remaining agent would not have represented an appropriate option. The decision to escalate corticosteroid doses was guided by the clinical grading of adverse events [30] and supported by existing literature, in which corticosteroids are commonly recommended for the management of lenvatinib-mediated hematologic toxicity and capillary leak–related complications [31]. Hematologic abnormalities improved, but CLS manifestations persisted. In light of this steroid-refractory course and guided by available literature [24,32,33], IVIG therapy was commenced, resulting in partial but clinically meaningful improvement, justifying maintenance with monthly infusions.

## 4. Conclusions

This case underscores the complexity of diagnosis and management of multisystem toxicities in patients receiving combined immunotherapy and TKI treatment for metastatic malignancies. Although the presence of a leukoerythroblastic reaction on peripheral blood smear in patients with advanced cancer commonly raises suspicion for bone marrow infiltration, alternative etiologies should also be carefully considered. In the present case, lenvatinib-associated thrombocytopenia, accompanied by marked erythropoietic stress, reflected by circulating nucleated red blood cells and dysplastic features, occurred concurrently with pembrolizumab-related CLS. Such overlapping treatment-related adverse events pose significant diagnostic challenges and require prompt recognition, comprehensive evaluation—including bone marrow examination—and timely discontinuation of the suspected agents. Careful clinical and laboratory monitoring remains essential following patient improvement, both to assess hematologic recovery and to detect potential delayed complications, including therapy-related myeloid disorders. Continued oncologic surveillance is equally important, given the underlying diagnosis of metastatic melanoma and the risk of disease recurrence.

## Figures and Tables

**Table 1:** Laboratory evaluation upon patient clinical presentation.

Test	Patient Result	Normal Range
White Blood Cells	5.66 × 10^9^/L	4.50–11.00 × 10^9^/L
Neutrophils	4.55 × 10^9^/L	1.55–6.50 × 10^9^/L
**Lymphocytes**	**0.75 × 10^9^/L**	1.20–3.40 × 10^9^/L
Monocytes	0.38 × 10^9^/L	0.10–0.90 × 10^9^/L
Eosinophils	0 × 10^9^/L	0.00–0.70 × 10^9^/L
Basophils	0.04 × 10^9^/L	0.00–0.20 × 10^9^/L
**Hemoglobin**	**10.9 g/dL**	12.0–16.0 g/dL
**Hematocrit**	**33.1%**	38.0–47.0%
Mean Corpuscular Volume	80.5 fL	80.0–96.0 fL
**Mean Corpuscular Hemoglobin**	**26.5 pg**	27.0–31.0 pg
Reticulocytes	107 × 10^9^/L	20–160 × 10^9^/L
**Platelets**	**22 × 10^9^/L**	140–440 × 10^9^/L
**Erythrocyte Sedimentation Rate**	**42 mm/h**	0–20 mm/h
INR	1.1	0.90–1.20
**aPTT**	**26 s**	29–40 s
Fibrinogen	279 mg/dL	180–400 mg/dL
AST	30 U/L	<32 U/L
ALT	32 U/L	<33 U/L
γGT	13 U/L	5–36 U/L
ALP	57 U/L	35–104 U/L
Troponin	6 pg/mL	<14 pg/mL
Thyroid-Stimulating Hormone	3.24 mIU/L	0.27–4.20 mIU/L
Free thyroxine	14.3 pmol/L	12.0–22.0 pmol/L
B-type natriuretic peptide	109 pg/mL	<115 pg/mL
**C-Reactive Protein**	**73 mg/L**	0–5 mg/L
**LDH**	**409 U/L**	135–214 U/L
**Albumin**	**27.6 g/L**	35.0–50.0 g/L
Iron	103 μg/dL	37–145 μg/dL
Ferritin	66 ng/mL	15–150 ng/mL
Vitamin B12	253 pg/mL	223–925 pg/mL
Folate	10.30 ng/mL	3.98–26.80 ng/mL
Haptoglobin	183 mg/dL	50–320 mg/dL
Direct Antiglobulin Test	Negative	Negative
Procalcitonin	Negative 0.06 ng/mL	0.02–0.50 ng/mL
Hepatitis B virus surface antigen	Negative 0.19 IU/mL	Negative
**Hepatitis B virus surface antibody**	**Positive 1.66 IU/mL**	Negative
Hepatitis B virus core antibody	Negative 0.06 IU/mL	Negative
Hepatitis C virus antibody	Negative 0.05 IU/mL	Negative
Human Immunodeficiency Virus antibody	Negative 0.17 IU/mL	Negative
**Cytomegalovirus IgG**	**Positive 82.80 U/mL**	<12 U/mL
Cytomegalovirus IgM	Negative <5 U/mL	<18 U/mL
**Viral capsid antigen Epstein–Barr Virus IgG**	**Positive 123 AU/mL**	<20 AU/mL
Viral capsid antigen Epstein–Barr Virus IgM	Negative <10 AU/mL	<20 AU/mL
Toxoplasma IgG	Negative <3.00 IU/mL	<7.20 AU/mL
Toxoplasma IgM	Negative <3.00 IU/mL	<6 IU/mL
Parvo-virus B19 IgG	Negative <0.10	0.9–1.1
Parvo-virus B19 IgM	Negative <0.10	0.9–1.1
Leishmania Antibody (Anti-K39)	Negative	Negative
Bartonella Antibody	Negative	Negative
Antinuclear Antibody	Negative	Negative (<1:80)
Anti-Parietal Cell Antibody	Negative	Negative
IFA	Negative	Negative
Serum Angiotensin-Converting Enzyme	Normal 45 U/L	40–50 U/L
Anti-cardiolipin IgG	Negative <31.0 U/mL	0–48 U/mL
Anti-cardiolipin IgM	Negative <31.0 U/mL	0–44 U/mL
Anti-β2GPI IgG	Negative <6.25 U/mL	0–9 U/mL
Anti-β2GPI IgM	Negative <6.25 U/mL	0–9 U/mL
Immunofixation by Electrophoresis	Negative	Negative
C3	167 mg/dL	90–180 mg/dL
**C4**	**47.9 mg/dL**	10.0–40.0 mg/dL
RF	<8.56 IU/mL	<20.00 IU/mL
**IgG**	**391 mg/dL**	700–1600 mg/dL
IgA	112 mg/dL	70–400 mg/dL
**IgM**	**<24.2 mg/dL**	40.0–230.0 mg/dL
**IgE**	**127 IU/mL**	0–100 IU/mL

Values beyond the reference range are shown in bold.

**Table 2:** A detailed chronological timeline of clinical events, treatments, and adverse effects.

Time Point	Clinical Event
13 years before	Primary melanoma diagnosed; surgical excision
4 years before	Metastatic progression
Month 0	Started nivolumab + ipilimumab
Month 3	Stable disease → nivolumab maintenance
Month 20	Disease progression
Month 21	Started lenvatinib + pembrolizumab
~2 years later	Complete metabolic response
3 months before	Treatment stopped due to grade 3 eosinophilia
3 to 0 months	Steroids (tapering)
Admission	Thrombocytopenia, generalized edema
Hospitalization	Exhaustive investigation—Steroid escalation—IVIG initiated
Follow-up	Monthly IVIG (4 cycles), dose of 20 g, stable patients

## Data Availability

The data presented in this study are available on request from the corresponding author.

## Abbreviations

The following abbreviations are used in this manuscript:

CLS	Capillary leak syndrome
CMR	Complete metabolic response
EPLIN	Epithelial protein lost in neoplasm
FGFR	Fibroblast growth factor receptors
ICI	Immune checkpoint inhibition
IL-	Interleukin-
INF-γ	Interferon gamma
IrAEs	Immune-related adverse events
IVIG	Intravenous immunoglobulin
NRBCs	Nucleated red blood cells
PAS	Periodic acid–Schiff
PDGFR	Platelet-derived growth factor receptor
RBCs	Red blood cells
SAAG	Serum–ascites albumin gradient
TKI	Tyrosine kinase inhibition
TNF-a	Tumor necrosis factor alpha
VE-cadherin	Vascular endothelial cadherin
VEGF	Vascular endothelial growth factor
WBC	White blood cells
